# The effects of carvacrol on the cardiac apoptosis gene expression levels in heart tissue of obese male rats induced by high-fat diet 

**DOI:** 10.22038/AJP.2024.25089

**Published:** 2025

**Authors:** Mahdi Ahmadi, Sadegh Bagherzadeh, Mohammad Hossein Boskabady, Ali Vahabi, Sakhavat Abolhasani, Mohammad Reza Aslani

**Affiliations:** 1 *Immunology Research Center, Tabriz University of Medical Sciences, Tabriz, Iran*; 2 *Department of Basic Sciences and Health, Sarab Faculty of Medical Sciences, Sarab, East Azerbaijan, Iran *; 3 *Student Research Committee, Tabriz University of Medical Sciences, Tabriz, Iran*; 4 *Applied Biomedical Research Center, Mashhad University of Medical Sciences, Mashhad, Iran*; 5 *Department of Immunology and Genetics, Faculty of Medicine, Urmia University of Medical Sciences, Urmia, Iran*; 6 *Lung Diseases Research Center, Ardabil University of Medical Sciences, Ardabil, Iran*; 7 *Department of Physiology, Faculty of Medicine, Ardabil University of Medical Sciences, Ardabil, Iran*

**Keywords:** High-fat diet, Obesity, Carvacrol, Apoptosis, Heart tissue

## Abstract

**Objective:**

Animal studies have revealed that lipid accumulation in obese mice fed with a high-fat diet (HFD) leads to alterations in the structural and functional properties of cardiovascular tissues. The current study aimed to investigate apoptosis/anti-apoptotic markers in the heart tissue of rats fed with a HFD.

**Materials and Methods:**

Twenty-four male Wistar rats (weighing approximately 180 grams) were randomly divided into three groups (n=8 each group), including the control group (C), the high-fat diet group (HFD), and the high-fat diet + carvacrol group (HFD + Carva). Animals received a standard or HFD to induce obesity for three months. From day 61 to 90 in the HFD+Carva group, carvacrol was injected intraperitoneally (50 mg/kg) every other day. At the end of the study, the heart tissue was examined for pathological changes and the mRNA levels of *TNF-α, Bcl2, Bax*, and *caspase3* in the heart tissue by Real Time-PCR.

**Results:**

HFD-induced obesity led to increased *TNF-α*, *caspase-3*, and *Bax* and decreased *Bcl-2* expression levels in heart tissue. Furthermore, histopathological changes in intracytoplasmic vacuole accumulation were evident in the HFD-obese animals. Carvacrol treatment significantly decreased the expression of *Bax*, *TNF-α*, and *caspase-3* and increased the expression of *Bcl-2* in heart tissue.

**Conclusion:**

In the findings, carvacrol was found to decrease the histopathological changes caused by HFD in heart tissue by suppressing the expression of genes involved in the apoptosis pathway.

## Introduction

Lifestyle changes and inactivity have made obesity one of the most critical health risk factors. Data from epidemiological research has demonstrated that  obesity, in addition to affecting the quality of life, has imposed a high socio-economic cost on societies (Calcaterra et al., 2020). Obesity and overweight are associated with various diseases such as type II diabetes, heart disease, metabolic syndrome, fatty liver, high blood pressure, respiratory disorders, and musculoskeletal disorders (Breuer et al., 2020; Minghelli et al., 2015). Chronic inflammation resulting from obesity is seen as a key factor in the onset of numerous diseases due to the release of specific cytokines and adipocytokines (Aslani et al., 2020; Ghobadi et al., 2019; Keyhanmanesh et al., 2018). The relationship between obesity and cardiovascular diseases has been reported in previous studies, although the exact pathophysiology mechanism is unknown (Avesta et al., 2022; Nejati et al., 2021). In addition, metabolic syndrome and diabetes have been reported to be risk factors for cardiomyopathy (Shabab et al., 2024). By releasing inflammatory factors, fat tissue provides the basis for pathological changes, reduced heart function, and arteriosclerosis (Cercato Fonseca, 2019). Apoptosis is programmed cell death that contributes to the homeostasis of the internal environment by removing old and damaged cells. However, disruption of apoptosis activity leads to inflammation and pathological cell death (Henson Tuder, 2008; Ulukaya et al., 2011). In an animal model of obesity caused by a high-fat diet (HFD), it has been revealed that the disruption of apoptosis/anti-apoptotic markers in heart tissue has led to cardiac damage (Pakiet A, 2020).

Recently, the focus has shifted towards using medicinal plants to treat a variety of diseases (Abedi et al., 2023; Aslani et al., 2022; Ghasemi et al., 2021; Khazdair et al., 2021; Saadat et al., 2021). In a review study, the protective effects of medicinal plants for cardiac disorders have been well reported (Shabab et al., 2021). Phenolic monoterpene compounds like carvacrol and thymol are present in plants like black cumin, oregano, sweet basil, thyme, and savoury. In traditional medicine, plants with high amounts of carvacrol and thymol have been used for therapeutic purposes such as stimulant, emmenagogue, anthelmintic, diuretic, carminative, anti-spasmodic, anti-microbial, reproduction, asthma, bronchitis, pharyngitis, emphysema, digestive system disorders, and cardioprotective (Patil et al., 2021; Rathod et al., 2021; Tariq, 2008). In an animal model of obesity, the protective effects of carvacrol have been revealed to be mediated by reducing the levels of the inflammatory cytokines released from adipose tissue, improving lipid profile levels, and reducing weight (Cho et al., 2012). In addition, the protective effects of carvacrol have been reported in doxorubicin-induced heart damage and the heart tissue of animal models of diabetes (Hou et al., 2019). Furthermore, after intervention with carvacrol, its cardioprotective effects have been observed through the reduction of myocardial infarct size, the increase of *superoxide dismutase* (SOD) and catalase (CAT) levels, and the reduction of cardiomyocyte apoptosis (Khajavi Rad et al., 2018).

Although the anti-inflammatory and antioxidant effects of carvacrol have been well demonstrated, its exact mechanism on apoptotic/anti-apoptotic markers in heart tissue in obese conditions is not clear. Induction of obesity with HFD has been of great interest in animal studies, which leads to changes in lipid profile, inflammatory, and histopathological markers in different tissues. The main focus of this investigation was to investigate the histopathological changes of the heart tissue and the expression of apoptosis/anti-apoptotic pathway genes (*Bax, Bcl-2,* and *caspase-3*) as well as inflammatory marker (*TNF-α*) in obese male rats induced by HFD.

## Materials and Methods

### Animals and diets

Twenty-four male Wistar rats (weight 180 g) were used in this study. One week after adaptation to the environment, the animals were divided into three groups (with 8 in each group) in a random manner as follows: control group (C): no intervention, HFD: animals were fed with HFD for three months, and HFD and carvacrol group (HFD + Carva): animals were fed with HFD for 12 weeks with intraperitoneal administration of carvacrol (50 mg/kg every other day for the last four weeks).

The basal diet used in the current animal study contained 11% fat, 22% protein, and 67% carbohydrate (Aslani, Keyhanmanesh, Khamaneh, Ebrahimi Saadatlou, et al., 2016). To induce obesity in animals, we used HFD with 32% fat, 20% protein, and 48% carbohydrates (Aslani et al., 2016).

### Real-time polymerase chain reaction

The heart's left ventricle was used to measure *TNF-α, Bax, caspase-3, *and* Bcl2* mRNA expression levels according to the method described in a previous study (Rahbarghazi et al., 2019). For this purpose, total RNA was isolated with an RNA extraction kit and subsequently quantified using a NanoDrop ND-1000 spectrophotometer (Thermo Scientific, Wilmington, DE, 19810 USA). Following this, cDNA synthesis was carried out by utilizing a cDNA synthesis kit. Each cDNA was used as a template during the process of quantitative real-time PCR, which involved the use of the SYBR Green master mix. *β-actin* was employed for normalizing the PCR products. Locked nucleic acid (LNA) forward and reverse primer sets (Exiqon) for mRNAs are displayed in Table 1. The findings are presented as a fold change.

### Histological examination

The impact of carvacrol on lessening cardiac tissue damage in obese male rats was carefully monitored over time. In order to achieve this goal, the right ventricles underwent fixation in a 10% buffered-formalin solution. Next, for pathological examinations, thin sections of the heart tissue (with a thickness of 4-5 microns) were prepared and stained with Hematoxylin-Eosin (H & E) solution. By utilizing a semi-quantitative scoring system, the degree of cardiac tissue damage in the final stage was determined through the accumulation of intracytoplasmic vacuoles. Accumulation of intracytoplasmic vacuoles in cardiac tissues was scored from 0 to 2 as follow: absence of pathologic injures =0; local injures =1; scattered injures =2 (Table 2). 

### Statistical analysis

The data is presented as mean±standard error of the mean (Mean±SEM). SPSS version 19 software was used to perform statistical calculations. The data obtained from the study were analyzed using One-Way ANOVA and Tukey's post hoc test. The criterion of the significance of the results in this study was p<0.05.

## Results

### Weight course

**Figure 1:**
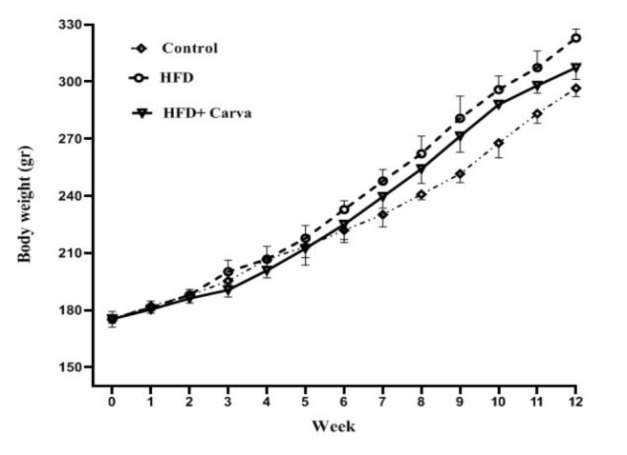
Weight courses in different groups. Values are expressed as mean±SEM. HFD: high fat diet, HFD+Carva: high fat diet and Carvacrol intervention. For each group, n=8.

Figure 1 shows weight course in the studied groups. After three months of obesity induction with HFD, it was found that weight gain was evident in the HFD and HFD+Carva groups compared to the control group (p<0.001 and p<0.05, respectively). Interestingly, in the HFD group, weight gain was significantly higher than the HFD+Carva group (p<0.01, Figure 1).

### Inflammatory marker expression levels in heart tissue

**Figure 2:**
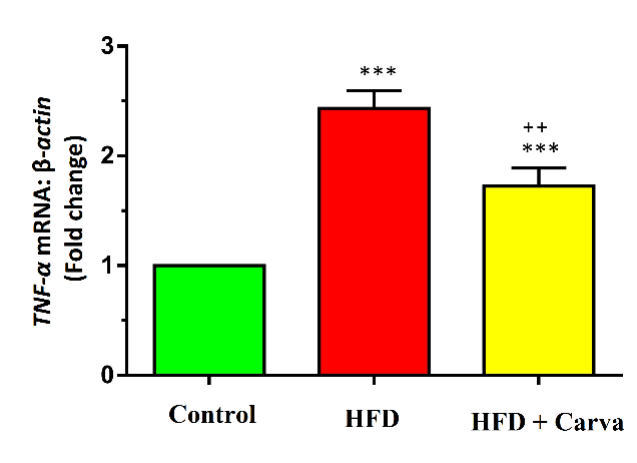
Heart tissue expression level of TNF-α in control group, HFD group, and HFD + Carva group. Values are expressed as mean+SEM. HFD: high fat diet, and Carva: carvacrol. ***p<0.001 control vs. other groups. ++p<0.01 HFD group vs. HFD + Carva group. Comparisons between groups were made using ANOVA test. For each group, n=8.

The increase in *TNF-α* levels in the heart tissue of both the HFD+Carva and HFD groups was markedly greater than the control group (p<0.001). *TNF-α* expression level was significantly reduced in the HFD+Carva group following treatment with carvacrol, as opposed to the HFD group (p<0.01, Figure 2).

### Apoptosis markers expression levels in heart tissue

**Figure 3:**
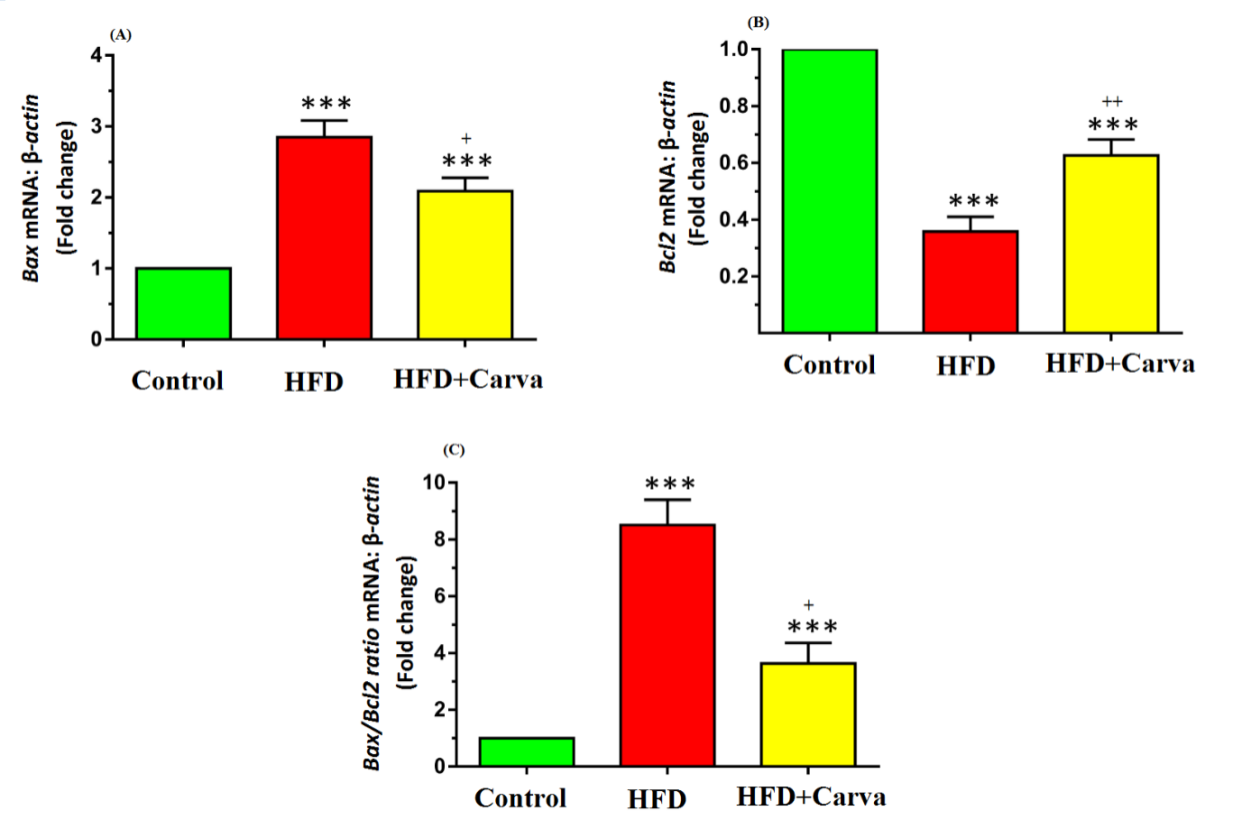
Heart tissue expression level of (A): Bax, (B): Bcl2, and (C): Bax/Bcl2 ratio in the control group, HFD group, and HFD + Carva group. Values are expressed as mean+SEM. HFD: high fat diet, and Carva: carvacrol. ***: p<0.001 control vs. other groups. +: p<0.05 HFD group vs. HFD + Carva group. Comparisons between groups were made using ANOVA test. For each group, n=8.

**Figure 4:**
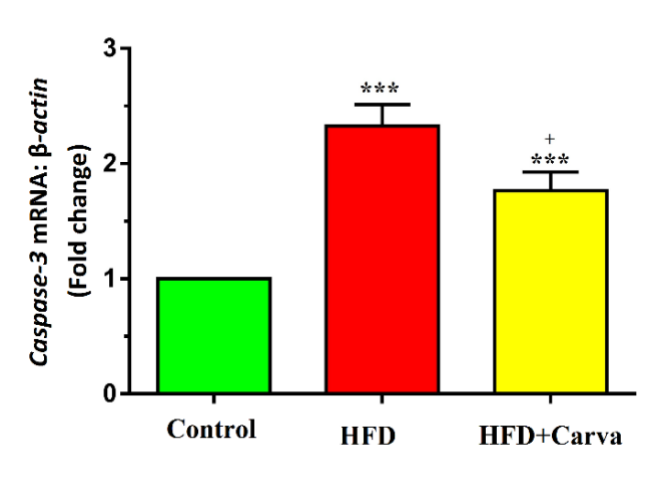
Heart tissue expression level of caspase-3 in the control group, HFD group, and HFD + Carva group. Values are expressed as mean+SEM. HFD: high-fat diet, and Carva: carvacrol. ***p<0.001 control vs. other groups. +p<0.05 HFD group vs. HFD + Carva group. Comparisons between groups were made using ANOVA test.For each group, n=8.

The expression levels of *Bax* (Figure 3A) and *caspase-3* (Figure 4) mRNA, along with the *Bax/Bcl-2* ratio (Figure 3C), were significantly higher in both the HFD+Carva and HFD groups compared to the control group (p<0.001). In the HFD+Carva group, carvacrol demonstrated a marked reduction in *Bax* and *caspase-3* mRNA expression, and the *Bax/Bcl-2* ratio compared to the HFD group (for all p<0.05, Figure 3A and C, and Figure 4).

On the other hand, there was a significant decrease in *Bcl-2* mRNA expression in both the HFD+Carva and HFD groups versus the control group (p<0.001). *Bcl-2* mRNA expression was significantly raised in the HFD+Carva group due to carvacrol treatment, as opposed to the HFD group (p<0.01, Figure 3B).

### Pathological change of Heart tissue

**Figure 5:**
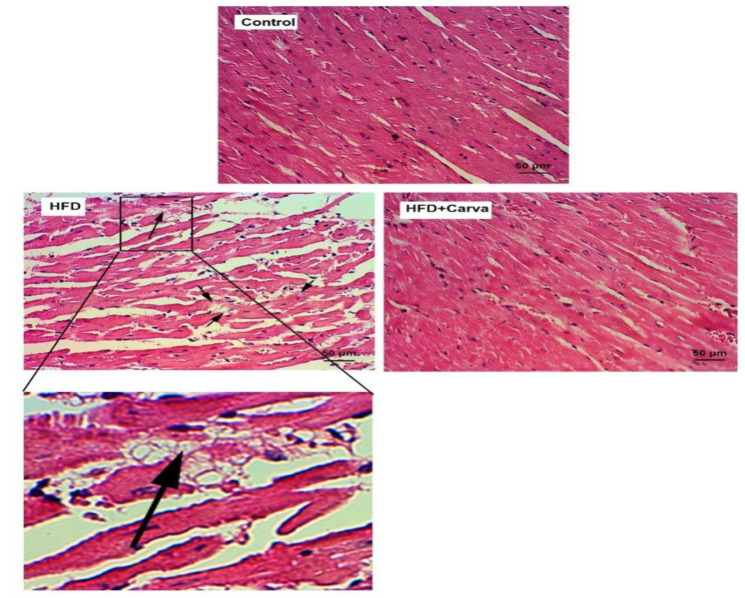
Pathological changes of the heart tissue of male rats in the control group, high fat-diet (HFD) group, and HFD+ carvacrol (Carva) group. Black arrows indicate the accumulation of intracytoplasmic vacuoles (watery degeneration) in heart tissue in the HFD group. Magnification 10 × 20. Staining was done with hematoxylin-eosin (H & E) solution.

In the HFD groups, pathological changes in the form of intracytoplasmic vacuoles accumulation (watery degeneration) were observed in the heart tissue (p<0.01 to p<0.05), and carvacrol in the group receiving high-fat diet + carvacrol (HFD + Carva) led to a decrease in pathological changes (p<0.01) (Figure 5 and Table 2). 

## Discussion

The male rats on a HFD exhibited elevated levels of *TNF-α* and apoptosis genes in their heart tissue, with increased *Bax* and *caspase-3* expression and decreased *Bcl2* expression. Treatment with carvacrol improved the inflammatory and apoptotic changes in the heart tissue of rats fed with HFD.

This study showed that feeding with a HFD in an animal model led to inflammatory (elevated *TNF-α* expression) and pathological changes (accumulation of intracytoplasmic vacuoles) in heart tissue. Studies have shown that obesity increases the production of inflammatory cytokines which play an essential role in causing cardiovascular disorders (Csige et al., 2018). Indeed, obesity is considered a low-

grade pro-inflammatory state (Akhavanakbari et al., 2019). Studies have shown that higher levels of pro-inflammatory factors as well as C-reactive protein (CRP) are linked to the development of obesity-induced cardiomyopathy (Trayhurn Wood, 2005) and apoptosis of cardiomyocytes (Chiong et al., 2011). In obesity, the increase of matrix metalloproteinases and the decrease of adiponectin, an anti-inflammatory adipokine, provide chronic inflammation for cardiac tissue damage (McGavock et al., 2006).

Evidence from the present study suggests that administering carvacrol to rats on HFD, can ameliorate inflammatory and pathological alterations in heart tissue. Carvacrol is known for its biological and medicinal properties, which include anti-apoptotic and anti-inflammatory effects (Suntres et al., 2015). Carvacrol treatment has been shown to result in higher levels of interferon-gamma (IFN-γ), lower levels of transforming growth factor beta (TGF-β), interleukin (IL)-4, and IL-17, and an improvement in serum levels of immunoglobulin E (IgE) (Khazdair et al., 2018). The observed therapeutic effects of carvacrol in conditions of HFD-induced obesity include reducing the levels of free fatty acids, triglycerides, and phospholipids (Khazdair et al., 2018), modulating inflammatory protein levels in adipose tissue (Cho et al., 2012), inhibiting adipogenesis (Cho et al., 2012), anti-hyperglycemic effects (Ezhumalai et al., 2014), and improving heart performance (Hou et al., 2019).

The current research discovered a reduction in *Bcl-2* expression in the heart tissue of animals on HFD. On the other hand, there was elevated expression of *Bax* and *caspase-3*, indicating a rise in apoptotic pathway activity. Increased apoptosis of cardiomyocytes has been reported in HFD-induced obesity with elevated DNA damage (Lee et al., 2007; Trivedi Barouch, 2008). Studies have shown that hyperglycemia and insulin resistance, as well as inflammatory cytokines released from adipose tissue, are factors that cause damage to cardiomyocytes and induce apoptosis (Schram Sweeney, 2008; Turer et al., 2012). Moreover, the presence of excess triglycerides in the hearts of obese people facilitates the formation of toxic substances like diacylglycerol and ceramide, leading to increased apoptosis in cardiomyocytes (Schram Sweeney, 2008). 

The results of this research indicate that carvacrol can suppress apoptosis in heart tissue through regulation of *caspase-3*, *Bcl2*, and *Bax* expression. In animal studies, the protective effects of carvacrol in heart tissue have been shown to be mediated by reducing collagen deposition in the myocardium, reducing the infarct area, and reducing oxidative stress (Chen et al., 2017). The anti-apoptotic properties of carvacrol have been shown to modulate the expression and protein levels of *Bcl-2, Bax,* and *caspase-3* in animal models with hypertrophic heart and acute myocardial infarction (Sadeghzadeh et al., 2018). The anti-apoptotic and protective effects of carvacrol have been shown in various *in vivo* and *in vitro* studies, such as human epithelial cells (Zeidan-Chulia et al., 2014), animal sepsis model (Ozer et al., 2017), focal cerebral ischemia (Yu et al., 2012), ischemia-reperfusion (I/R)-induced brain damage (Yu et al., 2012), cerebral ischemia damage (Chen et al., 2015), spinal cord injury (Jiang et al., 2015), I/R-induced kidney injury (Mustafa et al., 2011), testicular germ cell (Shoorei et al., 2019), thioacetamide-induced hepatocyte injury (Nafees et al., 2013), cadmium-induced lung injury (Yesildag et al., 2022), and I/R-induced liver injury conditions (Suo et al., 2014). 

Despite the anti-apoptotic evidence of carvacrol, some studies have revealed apoptosis-inducing effects in tumors and cancer cells. The inhibition of growth, proliferation, and cell migration in prostate cancer cells was achieved through the apoptotic effects of carvacrol (Keloushadi et al., 2015). It has been reported that carvacrol reduces the expression of caspase 3, 6, and 9 in MCF-7 cancer cells (Al-Fatlawi Ahmad, 2014; Arunasree, 2010).

Some of the limitations of the current study were 1- not determining the protein levels of cardiac apoptosis markers in the current study, 2- and not confirming the mechanistic effects of carvacrol through investigation of the inhibition of apoptotic pathways. Considering that various pathways are involved in the process of apoptosis, such as autophagy, mitophagy, and necroptosis, it is suggested that the effect of carvacrol on heart tissue in obesity induced by HFD be investigated on the mentioned pathways.

To conclude, the findings from this study revealed that HFD- induced obesity in rats resulted in heart tissue experiencing pathological changes, increased inflammatory cytokines, and an imbalance in apoptosis/anti-apoptotic markers. In obese rats fed with HFD, carvacrol was discovered to exhibit anti-inflammatory and anti-apoptotic characteristics that positively affected their heart tissue. 

However, further research is required to validate the impact of carvacrol on apoptotic mechanisms.

**Table 1:** Primers used in measuring gene expression

***Gene ***	**Primer sequence (** ***5*** **'-** ***3*** **')**	
	**Forward**	**Reverse**
*β-actin *	GGCACCACACCTTCTACAATG	GGGGTGTTGAAGGTCTCAAAC
*Caspase-3*	GCGGTATTGAGACAGACAGTGG	GCGCGTACAGTTTCAGCATGG
*Bax*	AAACTGGTGCTCAAGGCCCT	AGCAGCCGCTCACGGAG
*Bcl-2*	CCGGGAGAACAGGGTATGATAA	CCCACTCGTAGCCCCTCTG
*TNF-α*	GACCCTCACACTCAGATCATCTTCT	TGCTACGACGTGGGCTACG

**Table 2:** Pathological finding scores in the cardiac tissues of control group, HFD group, and HFD + Carva group.

**Pathological findings**	**Scores in groups (for each group, n = 6)**			
	**(Minimum-Maximum) **			
	C	HFD	HFD+Carva	
Accumulation of intracytoplasmic vacuoles	(0-0)	(1-2)**	(0-1)* ++	

Statistical differences between the control and HFD groups: *p<0.05 and **p<0.01. Statistical differences between the HFD and HFD + Carva groups: ++p<0. 01. The scales were placed inside the parentheses, representing the minimal and maximal pathological changes in each group.

## Authors' Contributions

MRA, and MA: Literature search, Proposal writing, Data collection, Analysis of data, Interpretation of data, Manuscript preparation, Review of manuscript.

SB, MHB, AV, and SA: Data collection, Interpretation of data, Manuscript preparation, Review of manuscript.
